# A better sequence-read simulator program for metagenomics

**DOI:** 10.1186/1471-2105-15-S9-S14

**Published:** 2014-09-10

**Authors:** Stephen Johnson, Brett Trost, Jeffrey R Long, Vanessa Pittet, Anthony Kusalik

**Affiliations:** 1 Department of Computer Science, University of Saskatchewan, 176 Thorvaldson Bldg., 110 Science Place, S7N 5C9 Saskatoon, Canada; 2 Department of Pathology and Laboratory Medicine, University of Saskatchewan, Room 2841, Royal University Hospital, 103 Hospital Drive, S7N 0W8 Saskatoon, Canada

**Keywords:** metagenomics, sequencing simulation, machine learning

## Abstract

**Background:**

There are many programs available for generating simulated whole-genome shotgun sequence reads. The data generated by many of these programs follow predefined models, which limits their use to the authors' original intentions. For example, many models assume that read lengths follow a uniform or normal distribution. Other programs generate models from actual sequencing data, but are limited to reads from single-genome studies. To our knowledge, there are no programs that allow a user to generate simulated data following non-parametric read-length distributions and quality profiles based on empirically-derived information from metagenomics sequencing data.

**Results:**

We present BEAR (Better Emulation for Artificial Reads), a program that uses a machine-learning approach to generate reads with lengths and quality values that closely match empirically-derived distributions. BEAR can emulate reads from various sequencing platforms, including Illumina, 454, and Ion Torrent. BEAR requires minimal user input, as it automatically determines appropriate parameter settings from user-supplied data. BEAR also uses a unique method for deriving run-specific error rates, and extracts useful statistics from the metagenomic data itself, such as quality-error models. Many existing simulators are specific to a particular sequencing technology; however, BEAR is not restricted in this way. Because of its flexibility, BEAR is particularly useful for emulating the behaviour of technologies like Ion Torrent, for which no dedicated sequencing simulators are currently available. BEAR is also the first metagenomic sequencing simulator program that automates the process of generating abundances, which can be an arduous task.

**Conclusions:**

BEAR is useful for evaluating data processing tools in genomics. It has many advantages over existing comparable software, such as generating more realistic reads and being independent of sequencing technology, and has features particularly useful for metagenomics work.

## Introduction

A common problem in metagenomic studies is that given real data (e.g., whole genome shotgun (WGS) sequences generated by next-generation sequencing (NGS) technologies), it is difficult to know if the bioinformatics analyses generate correct or complete results. In order to evaluate the results, the user typically needs to supply the bioinformatics programs (e.g., genome assembly software) with WGS sequencing data for which correct, complete results are known. As this is often not possible in the form of real sequencing data, it is instead necessary to use artificial reads generated *in silico*.

More generally, in the field of metagenomics there are few real datasets for which the correct results are known. Recent metagenomic studies of global algal distribution and human microbiome have derived results that conflict from previous studies in the same environments [1,2]. It is difficult to determine the usefulness of obtained results when their correctness is unknown. Even for problems such as *de novo *genome assembly, a simpler problem than metagenomic assembly, there is still debate as to which features make a "good" assembly due to significant variability in results between programs (e.g., high variability in average contig length and N50 values between programs) [3]. While some problem areas in bioinformatics such as multiple sequence alignment have resources like BAliBase for benchmarking, there are very few benchmarking datasets for metagenomics [4,5]. Furthermore, the simulated datasets used in previous metagenomic studies contain roughly 100 genomes, whereas actual metagenomic samples may have reads from thousands of organisms [6].

It would be far more convenient and accurate to simulate *in silico *NGS reads with known properties (correct outcomes), and subject that data to the analysis. For example, if a simulated-read dataset is generated based on completed genomes, then various assemblers can be evaluated by determining which assembler generates contigs best matching the original genomes. Such a basis for evaluation is preferable to traditional measures such as average contig length. For software pipelines, simulated data can provide insight with respect to optimal parameter settings. Unfortunately, read simulation is not as simple as selecting random subsequences from genomes. Read length, error rates, quality scores, and community abundances (for metagenomics) can have significant variation between samples. Thus, it is important to have a tool that can emulate all of these characteristics; the tool should generate artificial data that is as "messy" as real data.

Generating *in silico *NGS reads is not without difficulties. Each NGS technology has its own error rates, quality profiles, and read-length distributions (Illumina reads are generally uniform in length, reads from other technologies can vary greatly in length). Furthermore, the technologies are constantly improving in terms of generating longer, higher-quality reads. One can easily imagine developing software that mimics a given sequencing platform, and by the time the software is complete and tested, the platform has been significantly modified by its vendor. Another inconvenience of many modern sequencing simulator programs is that the user must determine appropriate settings for numerous parameters to generate data similar to real data. Exploring the parameter space can be a serious challenge, especially if documentation is sparse. Furthermore, modern sequencing simulator programs often have fixed, internal models for characteristics such as read length distributions and quality profiles. These models may not always reflect the characteristics observed in real reads. As such, a program designed for one type of sequencer (e.g., pyrosequencer) may not adequately simulate data from another (e.g. semiconductor sequencer). When sequencing simulator programs use these fixed models, they are generally limited to simulating a specific NGS technology.

To address these shortcomings, we have developed a software package called BEAR (Better Emulation for Artificial Reads). BEAR has, as input, a multi-FASTQ file (a file containing multiple sequences in FASTQ format) of WGS reads with the desired read length distribution and quality profile, as well as a source database. For metagenomics applications an abundance profile can be provided. BEAR generates simulated sequencing reads that are representative of genomes in the source database. The resulting data have a read length distribution and quality profile similar to those of the sample multi-FASTQ file. This approach allows for the emulation of read length distribution and quality profiles from various sequencing platforms. Since the artificial reads produced have known characteristics in terms of the source organisms and their correct assemblies, the data can then be used to evaluate techniques for analysis of NGS data (such as sequence assembly or community/diversity analysis in the case of WGS metagenomic data).

In this paper, we present our simulator and then compare it to five other popular sequencing simulator programs: Grinder [7], MetaSim [8], 454sim [9], SimSeq [10], and GemSIM [11]. Grinder and MetaSim are able to emulate Sanger, 454, and Illumina data, while 454sim and SimSeq are specific to 454 and Illumina, respectively. GemSim claims to be able to simulate any short-read simulator technology, but requires a reference genome for alignment of reads in order to derive its error model. We demonstrate that our program, BEAR, better emulates many features of WGS (meta)genomic reads from multiple NGS platforms without the need of a reference genome.

## Methods

### BEAR methodology

**Figure 1:**
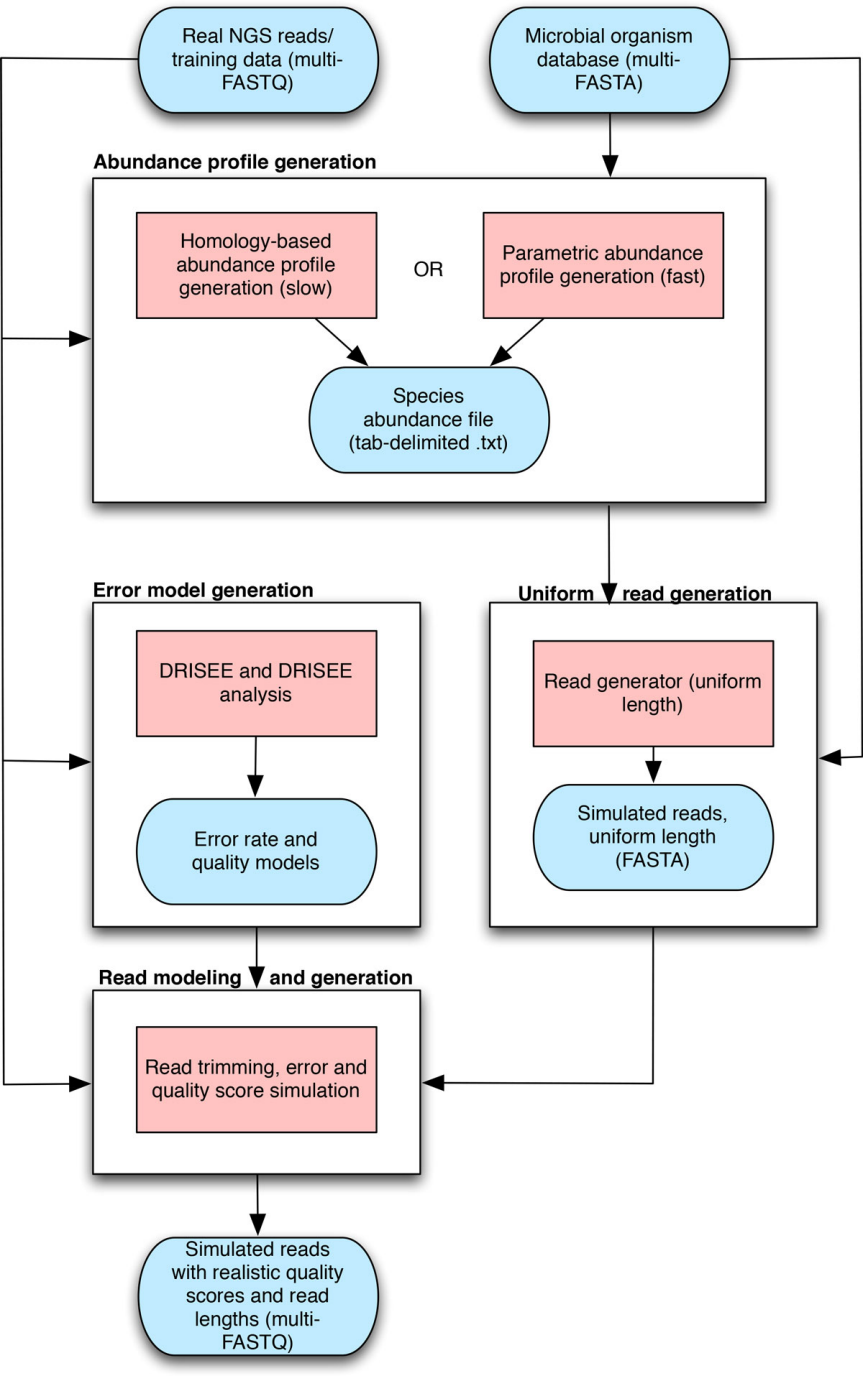
**BEAR workflow**. There are four major stages of using BEAR: error model generation, abundance profile generation, uniform read generation, and read modeling and generation. Blue rounded rectangles represent data files, red rectangles represent processes. Incoming and outgoing arrows represent input and output to and from processes, respectively.

Grinder, MetaSim, 454sim, SimSeq, and GemSIM were evaluated for their ability to emulate real data. Shortcomings identified in each of these programs were used to guide the development of BEAR. BEAR is implemented as a collection of Perl and Python scripts and available at https://github.com/sej917/BEAR The use of BEAR is free for academic purposes. A summary of the BEAR workflow is provided in Figure 1.

### Abundance profile generation

Abundance profile files are necessary input for existing metagenomic sequencing simulators. A common format for such files is that used by Grinder and GemSIM, which is tab-delimited text where the first column is a genome identifier, and the second column is the relative abundance of that genome in the simulated community. BEAR not only accepts abundance profiles in this format, but also provides users with the resources to generate them for any number of organisms. There are currently two separate methods for generating abundance profiles in BEAR:

• **Power function-based abundance profile generation (fast)**: This method derives abundance values from one of three power functions, where each function indicates either "low", "medium", or "high" species complexity. Plots of the three power functions are shown in Additional File 1 (Supplementary Data). A "low" species complexity represents an environment with few dominant species, while a "high" species complexity has no dominant species. The parameters of these functions were derived by fitting the abundance values of the simulated simLC, simMC, and simHC datasets from Pignatelli and Moya [12] to power functions.

• **Homology-based abundance profile generation (slow)**: This method derives abundance values by first determining the similarity of WGS shotgun reads in a user-supplied sample of real data to protein sequences (in genpept format) in the RefSeq database by using RAPSearch [13,14]. The accession number of each protein sequence is associated with taxonomy listed in a genpept record, allowing all significant hits in the WGS dataset to be associated with a taxonomy. The taxonomy of a given query (read) is calculated by taking all the lineages for all search results that have a bit score within a certain neighborhood of the highest one, finding the lowest common ancestor (LCA) among all those lineages. This approach is very similar to that of MEGAN [15]. The abundances of each species-level classification are then used to create the abundance profile.

### Error rate and quality-error model generation

BEAR uses both error rate and quality-error models when generating simulated data. The error rate model determines the probability of substitutions, insertions, or deletions at a given base pair position within a read. BEAR uses modified DRISEE scripts (included with BEAR) to infer error rates by first clustering artifactual duplicate reads in a user-supplied WGS dataset. DRISEE is a tool that bins all reads with identical 50bp prefixes, and processes each bin to predict error rates within the entire sample [16]. DRISEE then produces a file listing the substitution error rates for each nucleotide at each read position, and a combined insertion/deletion rate for each position. BEAR subjects all of these rates to exponential regression to create its error rate models. BEAR also analyzes the WGS input file and the DRISEE output file to determine the ratio of insertions to deletions at each position, and transition/transversion rates for substitutions.

The quality-error model determines the quality score to assign to a nucleotide resulting from a substitution or insertion error. This model is derived by processing the output from the modified DRISEE scripts and determining the average quality score assigned to erroneous nucleotides at each position in the read. BEAR performs second-degree polynomial regression on these values, creating a model for generating quality scores for incorrect base calls.

These error models are then used in the next stage to predict errors and quality scores for erroneous nucleotides.

If the user chooses not to use the DRISEE-derived error models, BEAR also has a lightweight default error model in which the probability of an incorrect base call is directly related to the predicted quality score at the current position in the simulated read. This simple model makes the assumption that substitutions, insertions, and deletions are all equally likely.

### Read modeling and generation

BEAR uses the abundance file and an organism database (a file containing the genomes) to generate a simulated dataset containing randomly sampled single or paired-end reads of uniform quality and length but reflecting the specified community composition. The organism database is in multi-FASTA format; i.e. it contains multiple sequences, each in FASTA form.

Next, input WGS reads are used as training data to create a read length distribution and a quality score model for correct base calls. The latter model is a position-dependent first-order Markov chain. That is, the quality score at a given position within a read influences the quality score at the next position. The script then uses the models for error rate, quality-error, read length distribution, and quality score along with the uniform-length reads to generate the final variable-length artificial reads.

More formally, we define a read of length *p *to be a string of characters *S *= *s*_1_*...s_p _*with an associated quality string *Q *= *q*_1_*...q_p_*. For each read, BEAR uses the training data to generate quality values *q_i_*, 1 *≤ i ≤ p *based on *q*_*i−*1 _and position *i − *1. In the case that *i *= 1, *q*_*i−*1 _= 0. Thus, for a given position *i *within a quality string *Q*, we wish to find *qi *by sampling from the conditional probability distribution *P *(*q_i_|q_i_−*1*, i − *1). This is only for producing nucleotides that are correct base calls. If an erroneous nucleotide is to be generated, the error model overrides the predicted quality value for *q_i_*. For example, in the case that the error rate model predicts a substitution error at position *i*, the Markov chain is not sampled and the quality-error model sets $q_{i}={a_{s}}_{i}i^{2}+b_{si}i+c_{si}$, where *a_s_i__, b_s_i__, c_s_i __*are the coefficients of the second-degree polynomial regression for the nucleotide *s_i_*.

### Testing

We compared BEAR to five sequencing simulator programs based on their ability to emulate the characteristics of actual sequence data obtained from Ion Torrent, 454, and Illumina sequencers. When determining the input organism databases and abundance files, we used the specific genomes and relative abundance values listed in previous work with simulated metagenomic data [12]. The programs that do not support abundance files were supplied with just the database of genome sequences. For each of the tested programs, parameters were chosen that would generate the read length and quality score distributions that most closely matched those of the actual test data.

### Training data

For each of the tests, a dataset was used to train BEAR. An Ion Torrent training set consisting of 377,630 raw metagenomic reads was generated using a Personal Genome Machine with an Ion 318 Chip. Another Ion Torrent training set consisting of 689,365 reads from the *E. coli *DH10B genome was used for comparing BEAR and GemSIM error models. A 454 training set consisting of 122,737 raw reads from Roche 454 Genome Sequence FLX platform and an Illumina training set of 14,376,051 reads were obtained from Pittet et al. [SRA: SRX216314] [17,18]. In the case of the Illumina dataset, only the first 100,000 reads were used. The sequence simulators were then evaluated by how closely they were able to emulate the characteristics of the training dataset.

## Results

Results of attempts to simulate NGS data with each program are provided in this section. A summary of our findings for the read length distributions, errors, and quality profiles for each of the tested sequencing programs can be found in Table 1. In general, most programs were only able to generate reads following a uniform distribution (454sim, SimSeq) or a normal distribution (Grinder, MetaSim). With respect to generating realistic quality profiles and error models, each program behaved differently. The parameters of 454sim were difficult to calibrate due to the lack of documentation explaining how the parameters affect the generated data. SimSeq was able to generate profiles that were high quality for the first 80bp and low/variable quality for the last 20bp. SimSeq's parameters do not appear to be empirically determined, but it has been used successfully for evaluating assemblies of Illumina data [3,10]. Grinder can generate data based on uniform, linear, and polynomial error models, but is only capable of generating two possible quality values per run (a "good" quality value for correct bases and a "bad" quality value for errors), which is highly uncharacteristic of raw reads. MetaSim provides a number of options for user-specified error parameters. Unfortunately, it did not support the generation of quality scores. GemSIM was able to generate non-parametric read length and quality score distributions. However, it can only derive error rates and quality scores by aligning reads to a reference genome. That is, GemSIM requires training on WGS reads from a single genome and therefore is unable to directly generate data having the error, quality, and read length characteristics of a given metagenomic sample.

**Table 1:** Summary of characteristics of read-length distributions and quality profiles for BEAR and popular sequencing simulator programs

Program	Read length distribution	Quality profiles	Errors
MetaSim	Uniform and Normal	Not generated	User-defined, parametric

SimSeq	Uniform	High quality for first 80bp, low quality after	User-defined, parametric

Grinder	Uniform and Normal	Binary; either "good" or "bad"	User-defined, parametric

454sim	Uniform	Highly sensitive to parameter settings	User-defined, parametric

GemSIM	Non-parametric	Non-parametric	Inferred from alignment to reference genome

BEAR	Non-parametric	Non-parametric for correct base calls, second-degree polynomial for errors	Inferred from log regression analysis of clustering artifactual duplicate reads within data

While we had each program generate three different simulated metagenomic datasets (simLC, simMC, simHC), the results for all three sets were indistinguishable in terms of the features (read length distribution, quality profiles) that were used for evaluation. Consequently, only the low complexity simulated dataset is shown in the figures.

### Read-length distributions

**Figure 2:**
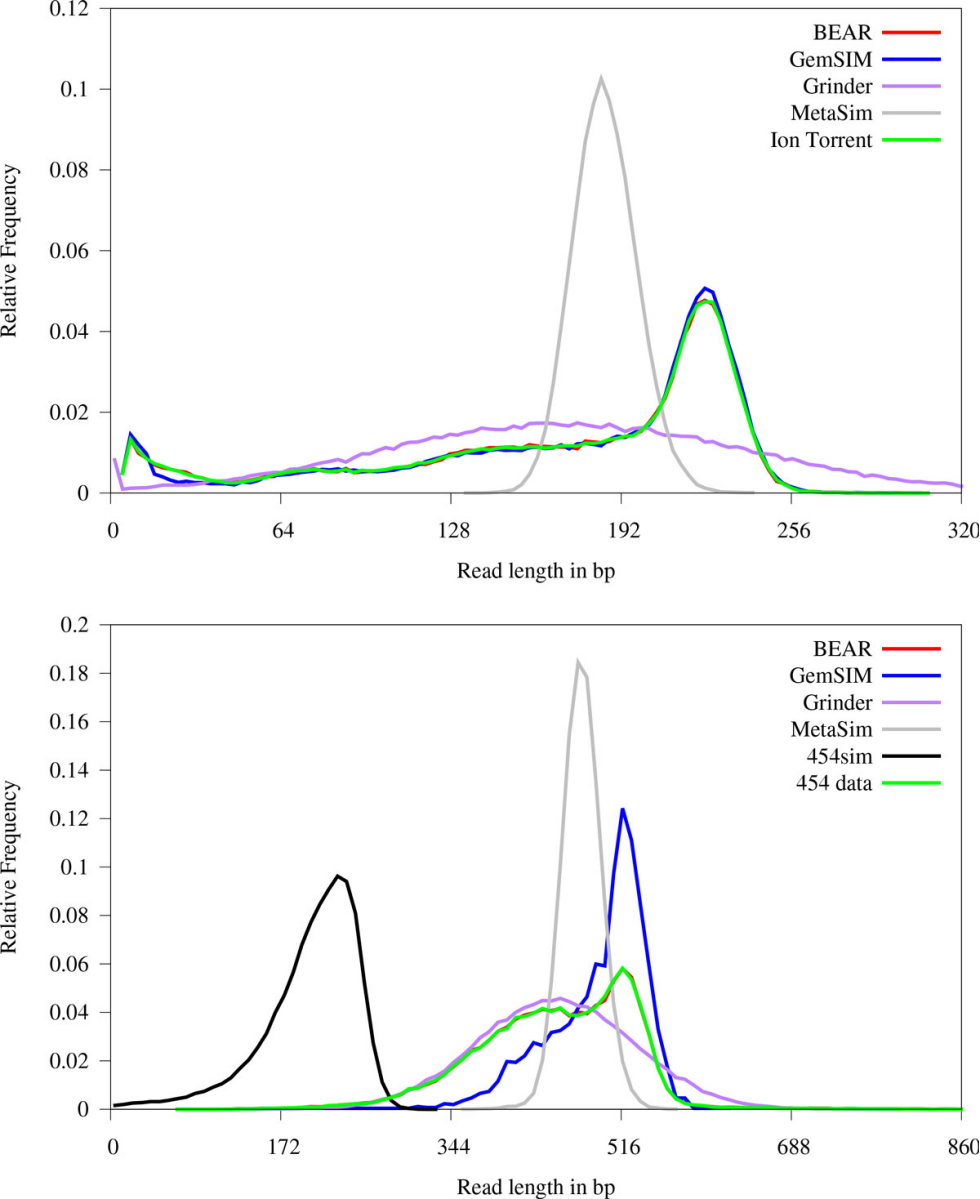
**Comparison of read length distributions generated by metagenomics sequencing simulator programs**. Top: Ion Torrent. Bottom: 454 data. When attempting to emulate these distribution, SimSeq only generated 100bp reads. Over 80% of the reads generated by 454sim were 165bp when emulating the Ion Torrent distribution, thus it is excluded from the top plot. Note that both panels BEAR closely matches the distributions of the real data. A panel for Illumina data is not shown since all real and simulated Illumina reads were 67bp in length.

The read-length distribution of the real WGS training data is compared to the distributions generated by each sequencing simulator program in Figure 2. As demonstrated in that figure, data obtained from actual NGS experiments is not necessarily simple enough to be characterized, for example, by supplying a mean and standard deviation of the read length distribution. GemSIM and BEAR are the only programs that closely modeled the Ion Torrent distribution. While the normally-distributed read lengths generated by Grinder and MetaSim model weren't as accurate as the read lengths generated by BEAR and GemSIM, they were far more accurate than those generated by 454sim and SimSeq. Over 80% of the reads generated by 454sim were 165bp, with read lengths never exceeding 175bp. SimSeq only generated reads 100bp in length. With respect to the 454 data, BEAR and Grinder matched the read length distribution far better than the other programs.

### Quality profiles

**Figure 3:**
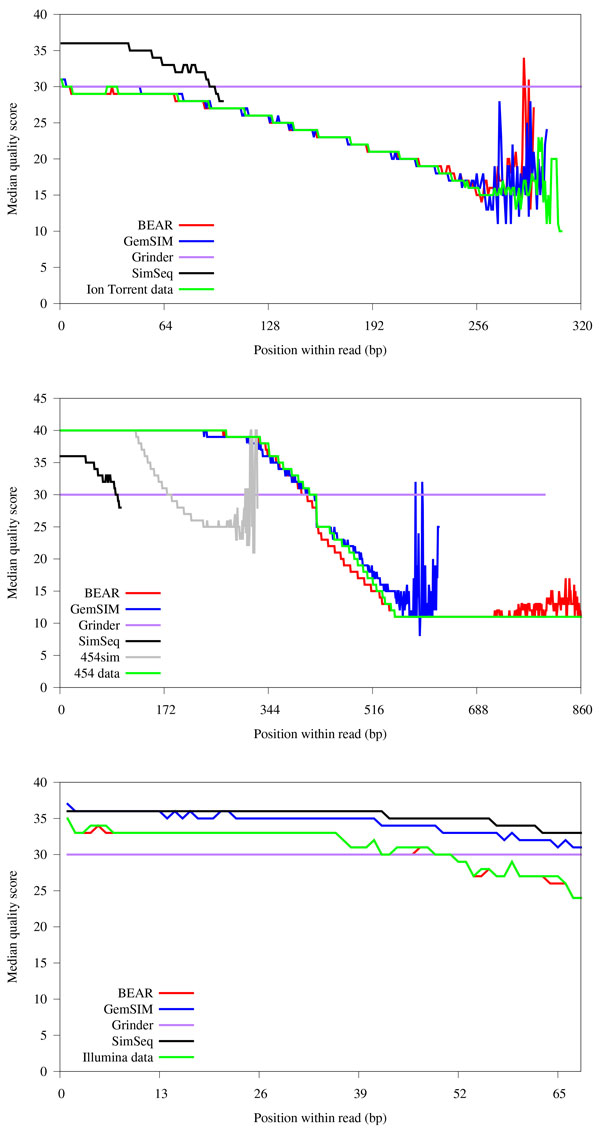
**Comparison of quality score distributions for real and simulated WGS datasets**. Top: Ion Torrent. Middle: 454. Bottom: Illumina. 454sim and MetaSim are excluded from the Ion Torrent and Illumina plots, as MetaSim does not generate quality scores and 454sim generated reads with a median quality score of 40 for all positions. GemSIM, 454sim, and SimSeq are unable to match the read length distribution of the 454 data, and as a result their quality score traces end prior to position 860.

Comparisons of quality profiles for all sequencing programs and WGS data can be seen in Figure 3. Similar to the read length distribution analysis, GemSIM and BEAR were the best of the tested simulator programs for generating data with the quality profile that most closely matched the real data. Near the end of the longer reads in the Ion Torrent and 454 data the base calls become quite noisy, leading to inconsistent quality values. While reads exceeding this length comprise a very small percentage of the data, it is worth noting that BEAR was able to generate noisy quality values after 250bp as well. Of SimSeq, Grinder, and 454sim, only SimSeq consistently produced non-constant quality scores. Unfortunately, it can only generate very short reads.

### Error models

**Figure 4:**
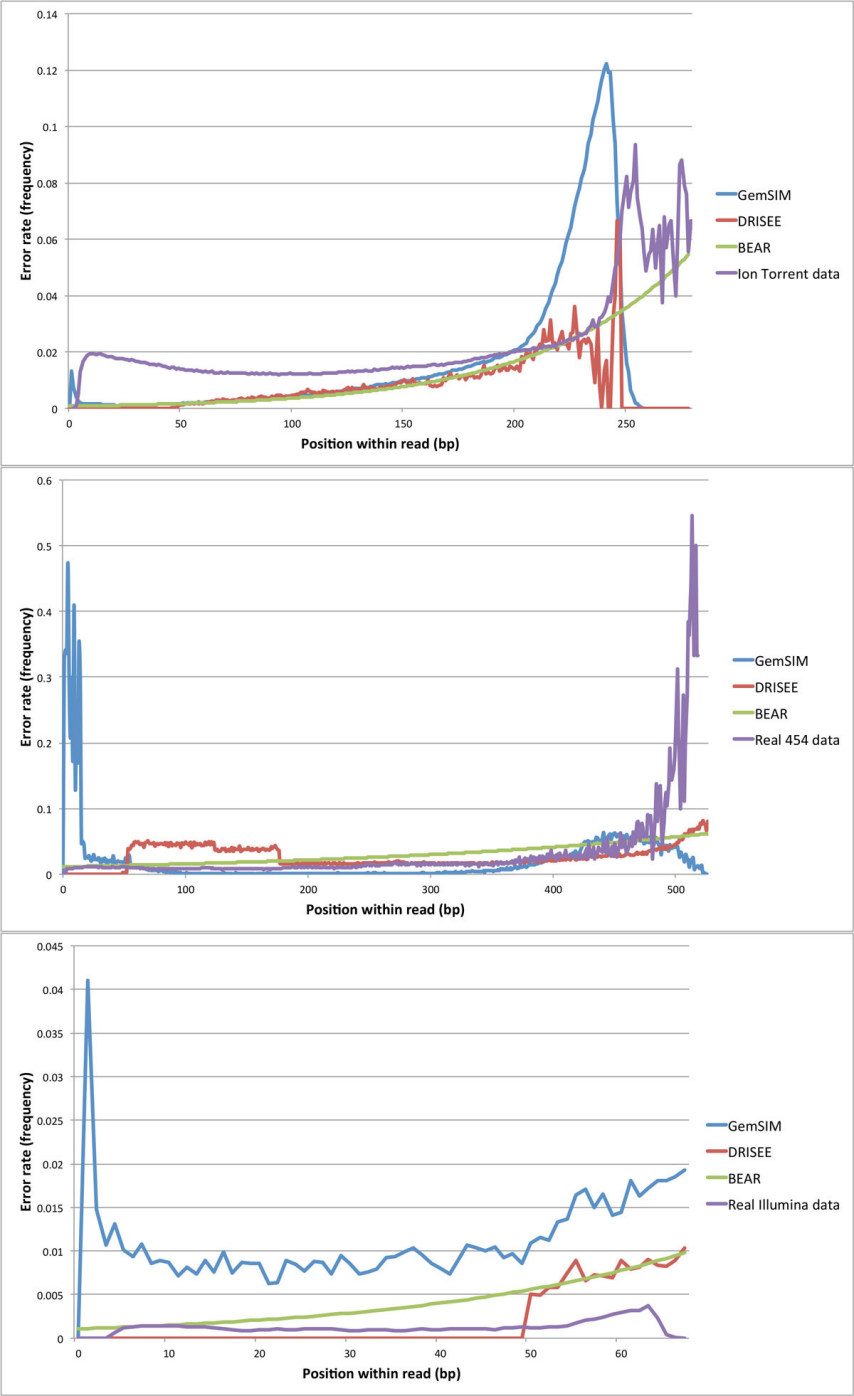
**Overall error rates for real WGS data and error rates predicted by GemSIM, DRISEE, and BEAR**. Top: Ion Torrent data; Middle: 454 data; Bottom: Illumina data. Real error rates were inferred by aligning reads to their respective reference genomes using Bowtie2 [19]. Error rates are displayed as relative frequencies.

Overall error rates predicted by GemSIM, DRISEE, and BEAR when supplied with various types of WGS data are compared in Figure 4. GemSIM failed to report error rates for every base pair position in the Ion Torrent and 454 datasets, in particular predicting error rates of 0 for positions beyond 250 and 525, respectively. In order to generate errors for all positions in long reads, BEAR automatically performs a exponential regression on the predicted error rates. This frees the user from the need for parameter tuning. This feature also allows BEAR to potentially generate substitution, insertion, and deletion errors at any possible read position, a feature that may not always be possible in GemSIM. GemSIM overestimated error rates at the beginning of all reads, and underestimated error rates at the ends of long reads (typically the most error prone region). GemSIM also overestimated the error rate at every position in the Illumina data. Conversely, BEAR predicted increases in error rates as read length increased for all datasets, with error models that more closely matched the real error rates.

BEAR also performs second-degree polynomial regression for determining the average quality score for an erroneous nucleotide. Plotting generated quality scores for erroneous bases and correct bases (data not shown) confirms that erroneous base calls generally have lower quality scores than correct base calls, two characteristics that are supported by the simultaneous decline in quality scores and increase in error rates observed in Figures 3 and 4, respectively.

## Discussion

The results above suggest that BEAR can be particularly useful for simulating raw genomic reads from NGS technologies such as Ion Torrent, which exhibit characteristics that most current programs are unable to emulate well. Figure 3 suggests that the general decline in median quality across the length of the read in actual Ion Torrent data is captured both by our position-dependent Markov chain-based approach, and the alignment-based context-dependent method used by GemSIM. However, for the 454 and Illumina datasets where the reads did not align as well to the reference genome, BEAR clearly emulated the read length and quality distributions better than GemSIM. In addition, BEAR can adapt to changes in NGS technology. For example, if the technology for a sequencing platform is modified to extend read length and quality characteristics, BEAR would be able to generate simulated data with these new qualities with no modifications. We also demonstrated that BEAR peforms well with both genomic and metagenomic data, exhibiting versatility that is lacking in other existing programs. Finally, we believe that BEAR belongs in a new category of sequencing simulator programs without the need for external parameter calibration. There are many possible extensions for BEAR, such as the generation of metatranscriptomic data, emulation of GC bias within a sample, paralellization, and development of more robust error models. However, further analysis and study needs to be completed before the results of any of these extensions can be reported.

## Conclusions

This paper presented BEAR, a tool for generating simulated reads based on empirically-derived read length distributions and quality scores. The approach used by BEAR for generating data eliminates the need for parameter tuning, allowing for an easy-to-use interface; the user need only provide a sample of data that has the desired properties of the reads to be emulated. We demonstrated that BEAR is superior to popular, existing artificial read generation programs in terms of producing reads with realistic read length and quality score distributions. While state-of-the-art programs such as GemSIM give comparable results in this regard, BEAR has additional features that make it more suitable for metagenomics applications, such as automatically producing community profiles and the lack of reliance on a reference genome. We believe that one of the best uses for BEAR will be for simulating metagenomic reads from emerging and consistently-updated technologies such as Ion Torrent, as there are few programs available that can capture their behaviour with respect to read length and overall quality scores. The features present in BEAR allow simulated data to be easily generated for analysis where one must know what the correct and complete output is.

## Competing interests

The authors declare that they have no competing interests.

## Authors' contributions

SJ wrote the code for BEAR except for the homology-based abundance file generation scripts, which were written by BT. SJ drafted the manuscript, JL, BT, VP, and AK revised it and contributed to the study design. AK supervised the work.

## Supplementary Material

Additional file 1 **Contains Figure S1, describing the power laws used for parametric abundance file generation**. Click here for file

## Declarations

This work was supported by MAVEN, a project funded by Western Economic Diversification Canada and Enterprise Saskatchewan, under the provisions of the Canada-Saskachewan Western Economic Development Partnership Agreement. Oversight of the MAVEN project is provided by Genome Prairie and Dr. Reno Pontarollo.

This article has been published as part of BMC Bioinformatics Volume 15 Supplement 9, 2014: Proceedings of the Fourth Annual RECOMB Satellite Workshop on Massively Parallel Sequencing (RECOMB-Seq 2014). The full contents of the supplement are available online at http://www.biomedcentral.com/bmcbioinformatics/supplements/15/S9.

## References

[B1] Worden AZ Janouskovec J McRose D Engman A Welsh RM Global distribution of a wild alga revealed by targeted metagenomics Current Biology 2012 22 17 R682 R683 10.1016/j.cub.2012.07.030 22974991

[B2] Gill SR Pop M DeBoy RT Eckburg PB Turnbaugh PJ Samuel BS Gordon JI Relman DA Fraser-Ligget CM Nelson KE Metagenomic analysis of the human distal gut microbiome Science 2006 312 5778 1355 1359 10.1126/science.1124234 16741115 PMC3027896

[B3] Bradnam KR Fass JN Alexandrov A Baranay P Bechner M Birol I Boisvert S Chapman JA Chapuis G Chikhi R Chitsaz H Chou WC Corbeil J Del Fabbro C Docking TR Durbin R Earl D Emrich S Fedotov P Fonseca NA Ganapathy G Gibbs RA Gnerre S Godzaridis E Goldstein S Haimel M Hall G Haussler D Hiatt JB Ho IYea Assemblathon 2: evaluating de novo methods of genome assembly in three vertebrate species GigaScience 2013 2 10 10.1186/2047-217X-2-10 23870653 PMC3844414

[B4] Thompson J Plewniak F Poch O BAliBase:A benchmark alignments database for the evaluation of multiple sequence alignment programs Bioinformatics 1999 15 87 88 10.1093/bioinformatics/15.1.87 10068696

[B5] Mavromatis K Ivanova N Barry K Shapiro H Goltsman E McHardy EC Rigoutsos I Salamov A Korzeniewski F Land M Lapidus A Grigoriev I Richardson P Hugenholtz P Kyrpides NC Use of simulated data sets to evaluate the fidelity of metagenomic processing methods Nature Methods 2007 4 495 500 10.1038/nmeth1043 17468765

[B6] Wooley JC Godzik A Friedberg I A Primer on Metagenomics PLoS Comp Biol 2010 6 2 e1000667 10.1371/journal.pcbi.1000667 PMC2829047 20195499

[B7] Angly FE Willner D Rohwer F Hugenholtz P Tyson GW Grinder: a versatile amplicon and shotgun sequence simulator Nucl Acids Res 2012 40 12 e94 10.1093/nar/gks251 22434876 PMC3384353

[B8] Richter D Ott F Auch AF Schmid R Huson DH MetaSim - A sequencing simulator for genomics and metagenomics PLoS One 2008 3 10 e3373 10.1371/journal.pone.0003373 18841204 PMC2556396

[B9] Lysholm F Andersson B Persson B An efficient simulator of 454 data using configurable statistical models BMC Research Notes 2011 4 449 10.1186/1756-0500-4-449 22029428 PMC3214204

[B10] Earl D Bradnam K St John J Darling A Lin D Fass J Yu HO Buffalo V Zerbino DR Diekhans M Nguyen N Ariyaratne PN Sung WK Ning Z Haimel M Simpson JT Fonseca NA Docking TR Ho IY Rokhsar DS Chikhi R Lavenier D Chapuis G Naquin D Maillet N Schatz MC Kelley DR Phillippy AM Koren Sea Assemblathon 1: A competitive assessment of short read assembly methods Genome Res 2011 21 2224 2241 10.1101/gr.126599.111 21926179 PMC3227110

[B11] McElroy KE Luciani F Thomas T GemSIM: general, error-model based simulator of next-generation sequencing data BMC Genomics 2012 13 74 10.1186/1471-2164-13-74 22336055 PMC3305602

[B12] Pignatelli M Moya A Evaluating the fidelity of de novo short read metagenomic assembly using simulated data PLoS One 2011 6 5 e19984 10.1371/journal.pone.0019984 21625384 PMC3100316

[B13] Pruitt KD Brown GR Hiatt SM Thibaud-Nissen F Astashyn A Ermolaeva O Farrell CM Hart J Landrum MJ McGarvey KM Murphy MR O'Leary NA Pujar S Rajput B Rangwala SH Riddick LD Shkeda A Sun H Tamez P Tully RE Wallin C Webb JD Weber amd Wu W Dicuccio M Kitts P Maglott DR Murphy TD Ostell JM RefSeq: an update on mammalian reference sequences Nucleic Acids Res 2013 10.1093/nar/gkt1114 PMC3965018 24259432

[B14] Ye Y Choi JH Tang H RAPSearch:a fast protein similarity search tool for short reads BMC Bioinformatics 2011 12 159 10.1186/1471-2105-12-159 21575167 PMC3113943

[B15] Huson DH Auch AF Qi J Schuster SC MEGAN Analysis of Metagenomic Data Genome Research 2007 17 377 386 10.1101/gr.5969107 17255551 PMC1800929

[B16] Keegan KP Trimble WL Wilkening J Wilke A Harrison T D'souza M Meyer F A platform-independent method for detecting errors in metagenomic sequencing data: DRISEE PLoS Comp Biol 2012 8 e1002541 10.1371/journal.pcbi.1002541 PMC3369934 22685393

[B17] Pittet V Ewen E Bushell B Ziola B Genome sequence of Lactobacillus rhamnosus ATCC 8530 J Bacteriol 2012 194 3 726 10.1128/JB.06430-11 22247527 PMC3264073

[B18] Pittet V Phister TG Ziola B Transcriptome Sequence and Plasmid Copy Number Analysis of the Brewery Isolate Pediococcus claussenii ATCC BAA-344T during Growth in Beer PLoS One 2013 8 9 e73627 10.1371/journal.pone.0073627 24040005 PMC3765258

[B19] Langmead B Salzberg S Fast gapped-read alignment with Bowtie 2 Nature Methods 2012 9 357 359 10.1038/nmeth.1923 22388286 PMC3322381

