# Supplementary material for: A better sequence-read simulator program for metagenomics
Source: BMC Bioinformatics. 2014 Sep 10;15(Suppl 9):S14. doi: 10.1186/1471-2105-15-S9-S14 (PMC4168713; doi:10.1186/1471-2105-15-S9-S14)
Supplement: Additional file 1 — Contains Figure S1, describing the power laws used for parametric abundance file generation. [file 1471-2105-15-S9-S14-S1.pdf]

Supplementary Data

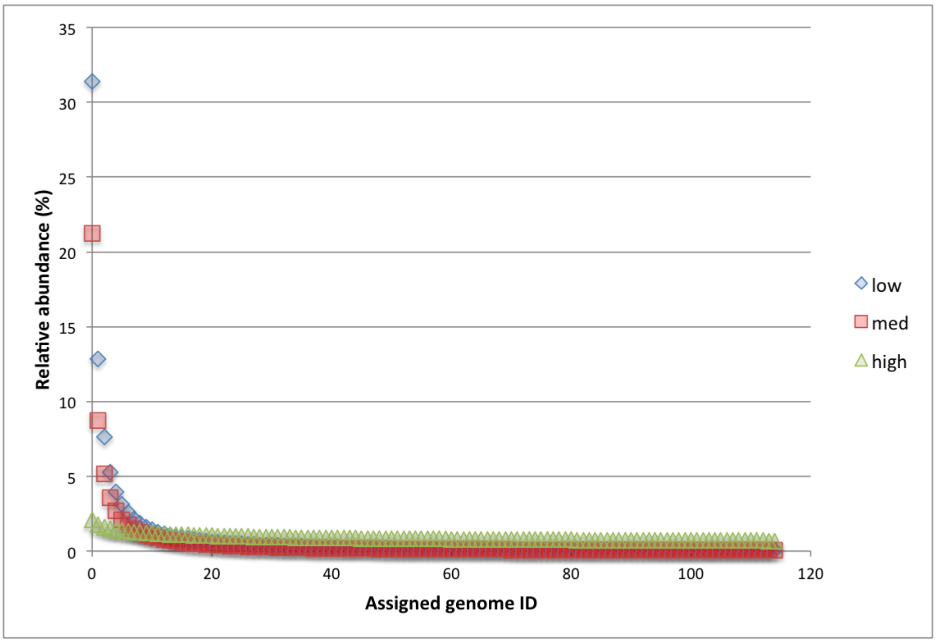

Figure S1: Abundance profiles derived from power functions. BEAR can generate three types of abundance profiles derived from power functions representing "low", "medium", or "high" species complexity. Low species complexity implies some dominant organisms in an environment, whereas high species complexity implies no dominant organisms. Genome sequences were sorted by their relative abundance in descending order and then assigned IDs from 1 to n, where n is the number of genome sequences in the environment.
